# A Single Talent Immunogenic Membrane Antigen and Novel Prognostic Predictor: voltage-dependent anion channel 1 (VDAC1) in Pancreatic Cancer

**DOI:** 10.1038/srep33648

**Published:** 2016-09-23

**Authors:** Weibin Wang, Taiping Zhang, Wenjing Zhao, Lai Xu, Yu Yang, Quan Liao, Yupei Zhao

**Affiliations:** 1 Department of General Surgery, Peking Union Medical College Hospital, Chinese Academy of Medical Science & Peking Union Medical College, 1 Shuai Fu Yuan Hu Tong, Beijing 100730, China; 2 Department of General Surgery, Beijing Tsinghua Chang Gung Hospital, Tsinghua University, 168 soup road, Changping District, Beijing 102218, China

## Abstract

Immunogenic membrane antigens associated with multiple biological functions of human cancer cells, have significant value in molecule diagnosis and targeted therapy. Here we screened immunogenic membrane antigens in pancreatic cancer by immunobloting IgG purified from sera of 66 pancreatic cancer patients with membrane proteins separated from two-dimensional PAGE of human pancreatic cancer cell line SWl990, and identified voltage-dependent anion channel 1 (VDAC1) as one of the potential immunogenic membrane antigens. Further studies focusing on VDAC1 demonstrated that VDAC1 mRNA and protein were significantly expressed in the tested pancreatic cancer cell lines. VDAC1 silencing with RNAi significantly decreased cell growth, invasion and migration in the pancreatic cancer cell line Capan-1. Additionally, VDAC1 expression was upregulated in pancreatic cancer tissue compared with normal pancreas samples and patients with low VDAC1 expression had a significantly greater median survival compared to those with high expression (27.0 months vs. 17.8 months, P = 0.039). In multivariable analysis, VDAC1 staining was an independent prognostic factor for survival [(Hazard-Ratio) HR = 1.544, 95% CI = 0.794–3.0, P = 0.021]. These results demonstrated that VDAC1 may be a candidate immunogenic membrane antigen for pancreatic cancer, a potential independent prognostic marker, and an ideal drug target.

Pancreatic cancer is a lethal malignancy with an extremely poor prognosis. The 5-year survival rate is approximately 6%, making it the fourth largest cause of cancer deaths in the United States^1^. Although the median survival time is 6 months, it differs among patients with different stages of the disease, ranging from 24.1 months for stage IA to 4.5 months for stage IV^2^. Patients with early-stage disease respond well to surgical interventions. Due to the lack of an effective means for early diagnosis, most pancreatic cancer patients are diagnosed at advanced stages with unresectable or metastatic disease. Therefore, more than 80% patients qualify only for palliative treatment. However, pancreatic cancer is resistant to chemotherapy, and gemcitabine-based multidisciplinary treatment as a predominant strategy for advanced pancreatic cancer has shown only a limited objective response^3^,^4^. In 2013, nab-paclitaxel plus gemcitabine revealed a significant survival benefit for phase 3 metastatic patients compared with gemcitabine (8.5 months vs. 6.7 months)^5^, but increased efficacy can only be achieved at the cost of more severe side effects. Therefore, we need to identify new molecules that play roles in pancreatic cancer to help better predict the progression of the disease and to potentiate target therapy to improve survival.

In pancreatic cancer, autoimmunity has been shown against several proteins, including MUC1, p53, and Rad51^6^,^7^,^8^. MUC1 is a transmembrane glycoprotein involved in cell-cell and cell-extracellular matrix interactions, and MUC1 autoantibodies have been observed in sera from patients with a variety of different tumors^9^. In pancreatic cancer, the presence of MUC1 IgG autoantibodies has been associated with a favorable prognosis^6^. The presence of p53 autoantibodies has been observed in 18.2% of patients with pancreatic cancer. However, p53 autoantibodies were also found in 5.3% of patients with acute pancreatitis and 12.1% of patients with chronic pancreatitis; therefore, the humoral response to p53 is not specific to malignancy. The recombination factor Rad51 is highly expressed in pancreatic adenocarcinoma, and Rad51 autoantibodies have been observed in 7% of patients with pancreatic cancer^10^. It is not clear why only a subset of patients with a particular tumor type develop a humoral response to that particular antigen. The identification of panels of tumor antigens that elicit immune responses may have utility in early cancer diagnosis, in establishing prognosis, and in immunotherapy against the disease. Several approaches are currently available for the identification of tumor antigens, in contrast to the identification of tumor antigens based on the analysis of recombinant proteins, employing a proteomic-based approach, such as that used here, for the identification of tumor antigens allows for the identification of autoantibodies against proteins as they occur in their natural states in lysates prepared from tumors and tumor cell lines.

Membrane proteins associated with pancreatic cancer perform many essential cellular functions, and the goal of this study was to screen and identify immunogenic membrane antigens in pancreatic cancer through membrane biology, cellular component proteomic, immuno-proteomic, and membrane proteomic approaches. Proteomic approaches were needed for the identification of tumor immunogenic membrane antigens that elicit humoral responses in the pancreatic cancer cell line SW1990. To this end, we used two-dimensional PAGE to simultaneously separate individual cellular membrane proteins from the SW1990 cell line. The separated membrane proteins were transferred onto polyvinylidene difluoride membranes. Serum IgG purified from clinically collected sera of pancreatic cancer patients, was used as the primary antibodies for the immunoblot. Membrane proteins specifically reacting with IgG antibodies from cancer patients were identified using mass spectrometry. Then, we checked the efficacies and studied the biological functions of the tumor-associated membrane candidates.

## Results

### Membrane protein 2-DE gel staining and positive dots of serum IgG via immunoblot

**Figure 1:**
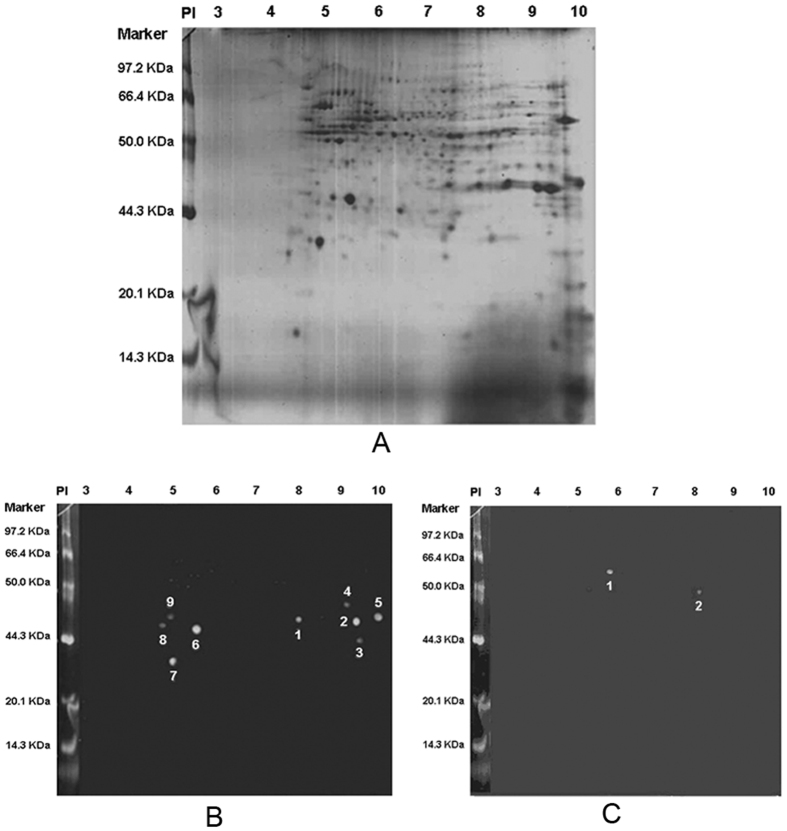
Pancreatic cancer cell SW1990 membrane protein expression and simulation of positive dots using a sera IgG immunoblot. (**A**) A total of 470 membrane protein dots were displayed using 2-DE with Coomassie brilliant blue staining. (**B**) In total, 9 positive protein dots were displayed using sera IgG from 66 pancreatic cancer patients when immunoblotting against SW1990 membrane proteins. (**C**) In total, 2 positive protein dots were displayed using sera IgG from 24 chronic pancreatitis patients when immunoblotting against SW1990 membrane proteins.

In total, 470 membrane protein dots of SW1990 cell were displayed on 2-DE by Coomassie brilliant blue staining. Then, using serum IgG purified from sera of 66 pancreatic cancer patients and 24 chronic pancreatitis patients to immunoblot with SW1990 cell membrane proteins, a total of 9 positive dots were identified from the pancreatic cancer patients, and 2 positive dots were identified from the chronic pancreatitis patients. However, there were no similar positive dots between the groups (Fig. 1).

### Identification of positive immunoblot dots by MALDI-TOF-MS

To identify reliable protein information, we conducted PMF of the 9 positive immunoblot dots with sera IgG of the pancreatic cancer patients using MALDI-TOF-MS and Mascot PMF Matching (Table 1). Among these positive immunoblot dots, VDAC1 was chosen for further investigation based on the following reasons: Firstly, three VDAC isoforms (VDAC1, VDAC2 and VDAC3) were identified from the 9 positive immunoblot dots^11^; Secondly, the VDAC family has been previously reported to be involved in regulating cell energy metabolism and apoptosis^12^; Thirdly, VDAC-1 has been identified as the most physiologically and metabolically important isoform in this family^13^.

### VDAC1 expression in normal and cancerous pancreatic tissues

**Figure 2:**
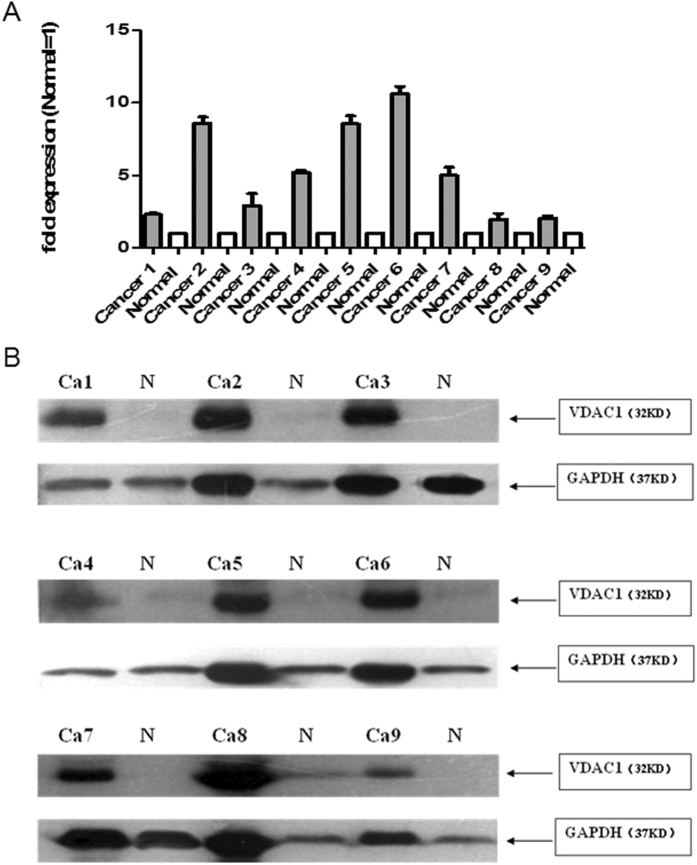
VDAC1 protein and mRNA expression in human pancreatic cancer tissue and normal pancreas. (**A**) VDAC1 protein expression in 9 paired human pancreatic cancer and normal pancreas. Immunoblot analysis was performed using anti-rabbit antibodies (1:1000). GAPDH was used as a loading control. (**B**) The expression of VDAC1 mRNA was analyzed in 9 paired human pancreatic cancer and normal pancreas using QRT-PCR analysis, the results were presented as the magnitude of relative expression (means ± SEM). β-actin was used as a housekeeping gene.

**Figure 3:**
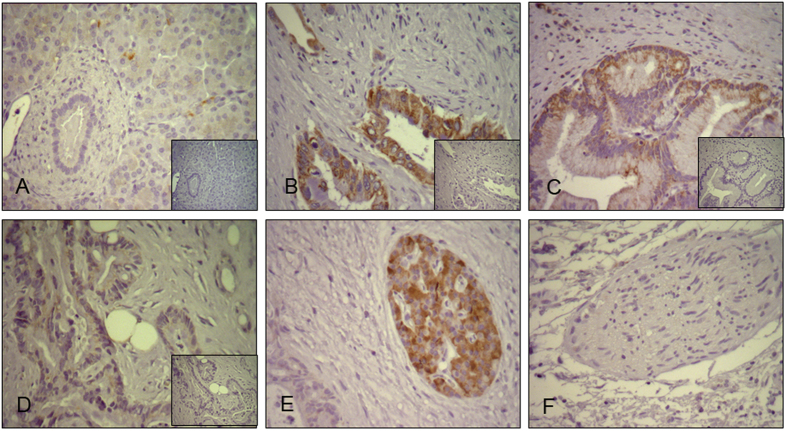
VDAC1 expression in normal and cancerous pancreatic tissues. Immunohistochemistry was performed with VDAC1 (1:5000) anti-mouse antibodies. (**A**) Localization of VDAC1 in normal pancreatic tissues, acinar cells and some ductal cells showed very weak cytoplasmic staining (**B**) Strong expression of VDAC1 shown by cytoplasmic staining of cancer cells. (**C**) Mild expression of VDAC1 shown by cytoplasmic staining of cancer cells. (**D**) Weak expression of VDAC1 shown by cytoplasmic staining of cancer cells. (**E**) Strong expression in pancreatic islet cells. (**F**) No staining in pancreatic nerves.

VDAC1 mRNA and protein were detected in 9 paired human pancreatic cancer tissues and normal pancreas (clinical characteristics in supplement). Results showed that VDAC1 mRNA and protein expression level in pancreatic cancer tissue were significant increased compared to normal pancreas samples respectively (Fig. 2). Semiquantitative immunohistochemistry was performed in 10 normal and 70 PDAC tissues (Table 2). In normal pancreas, acinar cells and some ductal cells showed very weak cytoplasmic staining (Fig. 3A). Strong VDAC1 expression was observed in cancer cells, degenerate acinar cells, while various intensities of VDAC1 positivity were observed in cancer cells from the PDAC tissue samples (Fig. 3B–D). In addition, VDAC1 was also strong expressed in pancreatic islet cells (Fig. 3E). The pancreatic nerves were generally VDAC1-negative (Fig. 3F). Obviously, the results of IHC revealed that the expression of VDAC1 was significantly higher in the PDAC tissues than in the normal pancreatic tissues.

Based on the VDAC1 different expression levels in PDAC tissues, we further divided the PDAC patients into VDAC1-weak and VDAC1-strong expression groups. The demographics and the correlation between VDAC1 expression and clinicopathological features are displayed in the Table 2. After the clinical and pathological survey, the results indicated the expression level of VDAC1 was not related to the number of patients, sex, age, TNM staging or histological grade.

### VDAC1 was expressed in PDAC cell lines, and VDAC1 silencing inhibited Capan-1 cell proliferation, invasion and migration

**Figure 4:**
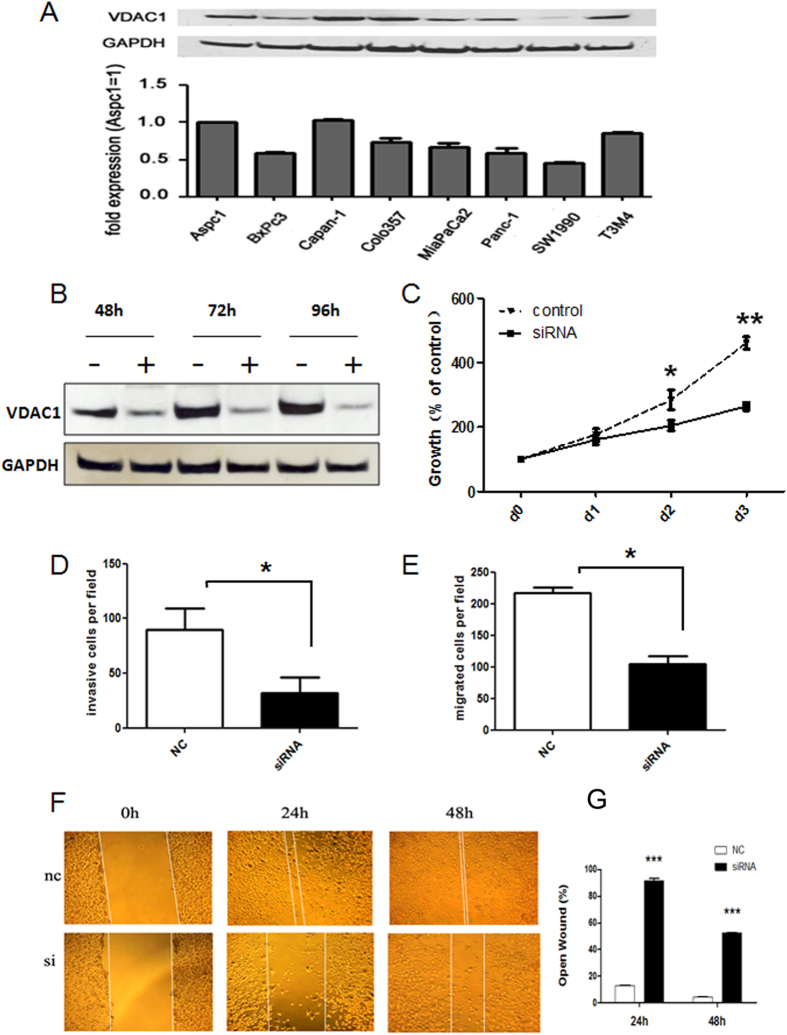
VDAC1 expression in human pancreatic cancer cell lines and the effect of VDAC1 on growth, invasion and migration. (**A**) VDAC1 protein and mRNA expression in eight human pancreatic cancer cell lines. Immunoblot analysis was performed using anti-rabbit antibodies (1:1000). GAPDH was used as a loading control. The expression of VDAC1 mRNA was analyzed in eight human pancreatic cancer cell lines using QRT-PCR analysis, and the results were presented as the magnitude of relative expression (means ± SEM). β-actin was used as a housekeeping gene. (**B**) The effect of RNAi on VDAC1 protein expression in Capan-1 cells (48 h, 72 h and 96 h). GAPDH was used as an internal control. (**C**) The effects of VDAC1 silencing on pancreatic cancer cell growth after 1, 2 and 3 days were assessed using a MTT assay (mean ± SD; *p < 0.05, **P < 0.01). (**D**) The invasiveness of Capan-1 cells was assessed after siRNA transfection (siRNA) and compared with negative control (NC)-transfected cells (mean ± SD; *p < 0.05). (**E**) The migration of Capan-1 cells was assessed after siRNA transfection (siRNA) and compared with negative control (NC)-transfected cells (mean ± SD; *p < 0.05). (**F**–**G**) Effects of VDAC1 silencing on pancreatic cancer cell migration were assessed using a wound-healing migration assay after 0 h, 24 h and 48 h (mean ± SD; ***P < 0.001)).

To investigate the biological function of the VDAC1 protein in pancreatic cancer, we firstly detected its expression in eight pancreatic cancer cell lines (Fig. 4A). The results showed that VDAC1 mRNA and protein were both detectable in all of these cell lines, and the Capan-1 cell line was chosen for further functional experiments due to its high level of VDAC1 expression.

Then, we conducted transient silencing of VDAC1 in the Capan-1 cell line. As expected, transfection with specific siRNA molecules resulted in 54%, 77% and 87% reductions of VDAC1 protein in the Capan-1 cells at 48 h, 72 h and 96 h, respectively (Fig. 4B). MTT assay was performed to assess the effects of VDAC1 silencing on proliferation in Capan-1 cells. We observed a significantly decreased growth rate after the effective reduction of endogenous VDAC1 compared with the control (Fig. 4C). Transwell assays were performed to examine the effects of VDAC1 silencing on cell invasion and migration in the Capan-1 cells. We observed that the silencing of VDAC1 resulted in a 67% and 71% reduction of cell invasion and migration ability respectively (P < 0.05, Fig. 4D,E). Furthermore, we validated VDAC1 silencing obviously inhibited cell migration ability in wound-healing migration assay performed at 24 and 48 hours compared with the control (P < 0.001, Fig. 4F,G).

### Correlation of VDAC1 expression in pancreatic cancer with Survival and Multivariable analysis of prognostic value of VDAC1 expression in pancreatic cancer

**Figure 5:**
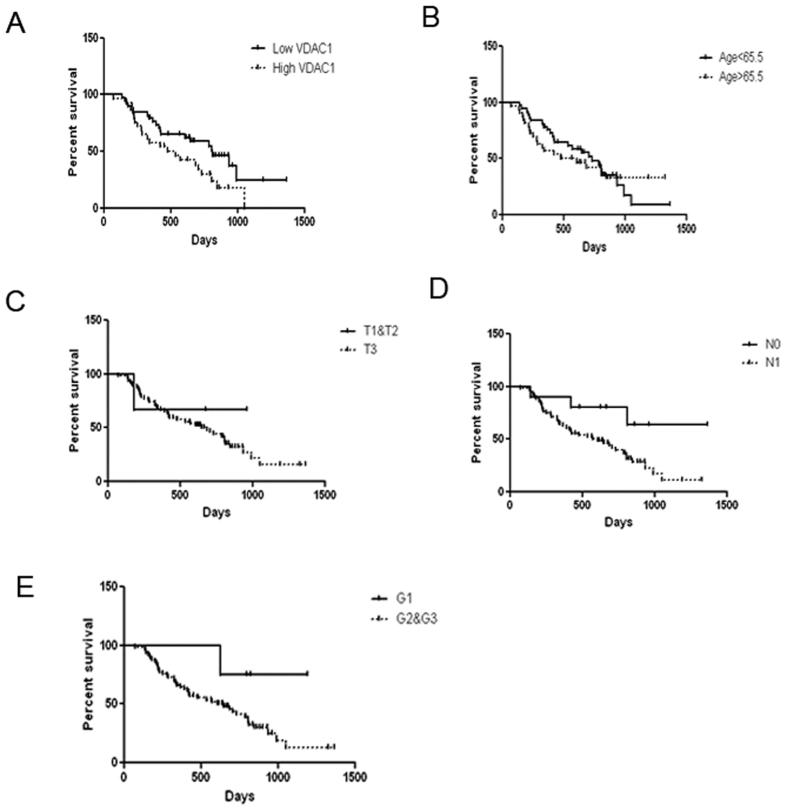
Kaplan-Meier and log-rank analyses of prognostic factors assessed in a multivariable analysis using a Cox proportional hazards model. Median values were taken as cut-off limits when the two groups were compared. Survival analyses were performed using the Kaplan-Meier method for the estimation of event rates, and a log-rank test was used for survival comparisons between patient groups. The P value was 5%.

Patients with lower levels of VDAC1 expression [n = 38, median survival = 27.0(19.1–34.9) months] had significantly longer survival times compared to those with higher VDAC1 expression [n = 29, median survival = 17.8(8.5–27.1) months, p = 0.039] (Fig. 5A). The prognostic value of VDAC1 expression was assessed by multivariable analysis using a Cox proportional hazards model (Table 3). By the factors analyzed, the results revealed that the expression of VDAC1 (HR = 1.544, 95%CI:0.794–3.0,P = 0.021) and lymph node metastasis (HR = 5.375, 95% CI:1.225–23.584,P = 0.026) both were independent prognostic factors (Fig. 5A,D). Because of the scarcity of early and less aggressive tumors in the cohort of 70 patients, other known prognostic factors (T-status, Grade)-although showing the classical tendencies-did not appear to impact on the prognosis independently (Table 3 and Fig. 5B–E).

## Discussion

VDACs, also known as mitochondrial porins, are located in the outer membranes of mitochondria and in the plasma membrane^11^,^14^. In vertebrates, three VDAC isoforms encoded by three separate genes have been characterized: VDAC1, VDAC2 and VDAC3^12^. The three isoforms are highly conserved and share a common signature in their C-terminal region, and all of them can be found in most tissues at varying amounts, with the most abundant form being VDAC1 and the least abundant form being VDAC3^15^. Among the VDAC family members, VDAC-1 has been identified as the most physiologically and metabolically important isoform. The structure of VDAC1 is characterized by a 19-stranded β-barrel and a 25-residue-long N-terminal α-helical region^16^.

VDAC1 acts as a channel in the outer mitochondrial membrane, and the pore formed through the outer mitochondrial membrane (OMM) by VDAC1 is almost freely permeable to low molecular weight molecules. This demonstrates that VDAC1 regulates metabolic cross-talk and energy exchange between mitochondria and cytosol, including mediating transport of metabolites, pyruvate, anions, cations, ATP and NADH^12^,^17^,^18^. Moreover, VDAC1 is also involved in mitochondria-mediated apoptosis and apoptosis regulation. Studies have shown that VDAC1 plays an essential role in the release of apoptosis proteins during the early stages of the apoptotic process and in interactions with proteins such as Bax, Bak and Bcl-xL^13^,^19^,^20^. Interfering with the interaction between BCL-xL and VDAC1 can lead to an increase in apoptosis and is beneficial for chemotherapy^21^. In addition, due to the particular boundary location of VDAC1, it can interact with proteins, including hexokinase (HK), tubulin, and actin, and thus mediate the integration of mitochondrial function with cellular activities^12^. By specifically binding to VDAC1, HK provides both a metabolic benefit and inhibition of apoptosis, and disruption of this interaction could be a strategy for inducing cell death.

Due to its key role in regulating cell life and death, studies have associated VDAC1 with cancer. As a dynamic regulator of metabolism, VDAC1 has been associated with a metabolic phenotype in cancer cells^22^. Studies have shown that VDAC1 is overexpressed in many cancer types, and VDAC1 gene expression has been identified as a predictor of poor outcomes in non-small cell lung cancer. VDAC1-associated genes can predict recurrence-free survival in many cancers^13^,^23^,^24^. Silencing VDAC1 expression in cancer cells inhibits tumor development *in vivo*^25^; conversely, high VDAC1 levels increase tumor cell energy-dependent processes, including proliferation and invasiveness^26^. In addition, VDAC1 is required for apoptosis induction. Tajeddine *et al.* reported that efficiently silencing VDAC1 inhibited cisplatin-induced apoptosis and Bax activation in non-small cell lung cancer^27^.

Performing IHC assays on 70 pancreatic cancer tissues and 10 normal pancreas samples, our present study demonstrated that VDAC1 levels were significantly higher in pancreatic cancer tissues, which was consistent with previous reports^24^. We also found that greater VDAC1 expression predicted a shorter overall survival time based on survival analysis, suggesting that VDAC1 could serve as a potential prognostic factor for survival. Similar results were reported in a study by Claire and colleagues, who demonstrated that VDAC1 gene expression is a predictor of poor outcome in NSCLC^23^.

Furthermore, we conducted biological function experiments to down regulate VDAC1 protein levels in the human pancreatic cancer cell line Capan-1 using transient transfection. VDAC1 silencing inhibited the growth, invasion and migration of Capan-1, suggesting its function in energy metabolism, which was consistent with the report from Tajeddine^27^. According to previous studies, due to its key role in metabolic and apoptotic regulation, VDAC1 is also involved in pancreatic cancer progression, is being studied as a potential anti-cancer therapeutic target^15^,^28^.

In summary, we identified VDAC1 as a candidate immunogenic membrane antigen of pancreatic cancer. The VDAC1 expression was upregulated in PDAC tissue and correlated with poor prognosis. VDAC1 played a role in modulating malignant phenotype in pancreatic cancer cells, could serve as a prognostic predictor and a candidate for targeted therapy against pancreatic cancer.

## Materials and Methods

### Cell lines

Eight human pancreatic cancer cell lines, including AsPC-1, BxPC-3, Capan-1, Colo-357, MIA PaCa-2, Panc-1, SU86.86 and T3M4, were kindly donated by Professor Helmut Freiss from the Technical University Munich of Germany. All of the cell lines were individually cultured in a humidified incubator with 5% CO2 at 37 °C in either RPMI-1640 medium or Dulbecco’s modified Eagle’s medium (DMEM, Hyclone, Thermo Fisher Scientific Inc., Waltham, MA) containing 10% fetal bovine serum (FBS; Hyclone).

### Clinical samples

All sera and pancreatic tissues were obtained from the Department of General Surgery, Peking Union Medical College Hospital (PUMCH), China. Sample collection and preservation were performed as described previously. The experimental protocol was approved by the Ethics Committee of PUMCH and written informed consent was obtained from all participants. The present study was conducted in accordance with the guidelines of the Declaration of Helsinki.

### Cellular Membrane Protein Extraction

We collected SW1990 cells using a ProteoPrep Membrane Extraction Kit (Sigma), after which the cells were suspended in a low conductivity buffer and disrupted by ultrasonication and other appropriate methods. The membranes and membrane proteins were centrifuged, washed with water and resuspended in a chaotropic buffer solution. This protein solution was then reduced and alkylated. The end result was a soluble membrane protein sample that contained low salt levels and was ready for separation using isoelectric focusing (IEF). After extraction, the membrane proteins were immediately frozen at −80 °C.

### Sera IgG Purification

We used a PROSEP-G kit (Millipore, Bedford, MA) to extract and purify sera IgG antibodies from 66 pancreatic cancer patients and 24 chronic pancreatitis patients. During the experiment, we collected eluent, extracted a sample using 12% SDS-PAGE, and scanned the Coomassie blue-stained gel to check the concentration and effectiveness of the extracts.

### 2D SDS-PAGE and Western Blot Analysis

Membrane proteins derived from the cultured cells were subjected to two dimensional electrophoresis as described previously^29^. In brief, solubilized membrane proteins were applied to isoelectric focusing gels. Isoelectric focusing was performed using pH 3–10 carrier ampholytes at 150 V for 4 h, 500 V for 1 h, 1000 V for 1 h and a gradient at 8000 V for 1 h and 8000 V for 12 h. The first-dimension gels were loaded onto three parallel second-dimension gels after equilibration in 1% DTT and 2% IAA. For the second dimension separation, a gradient of 11–14% acrylamide (Crescent Chemical, Hauppauge, NY) was used. The membrane proteins on the two gels were transferred onto an Immobilon-P polyvinylidene difluoride membrane (Millipore), and the third gel was visualized using Coomassie staining. After transfer, the membranes were incubated with a blocking buffer consisting of 10 mM Tris-HCl (pH 7.5), 50 mM NaCl, 1.8% nonfat dry milk, and 0.01% Tween 20 for 2 h. The membranes were incubated for 3 h at room temperature with IgG antibodies obtained from the sera of pancreatic cancer and chronic pancreatitis patients at a 1:1000 dilution. After three washes with washing buffer (Tris-buffered saline containing 0.01% Tween 20), the membranes were incubated with horseradish peroxidase-conjugated sheep antihuman (Amersham Biosciences, Piscataway, NJ) IgG antibodies at a dilution of 1:5000 for 1 h at room temperature. Immunodetection was accomplished using enhanced chemiluminescence (Amersham Biosciences) followed by autoradiography. We compared the positive dots to the parallel Coomassie-stained gel.

### Gel Enzyme Digestion and Mass Spectrometry

Protein identification using peptide mass fingerprinting (PMF) was carried out as previously described. After visualization with Coomassie blue staining, the electrophoretic spots were excised, destained with a solution of 5 mM ammonium carbonate/50% acetonitrile, and dehydrated with acetonitrile. The proteins were digested overnight at 37 °C with 12.5 ng/μl trypsin, and 0.75 μl of each supernatant was directly spotted onto the target disk and air-dried. Then, 0.75 μl of the matrix solution (a saturated solution of α-cyano-4-hydroxycynnamic acid in 50% v/v acetonitrile/0.5% v/v TFA) was added to the dried samples and again allowed to dry. The peptides were then concentrated using C14 pipette tips (Millipore). Spectra were obtained using a MALDI-TOF mass spectrometer (Amersham Biosciences). A PMF database search was carried out using PePtldent (www.expasy.org) and MASCOT (www.matrixscience.com) online software.

### RNA extraction and quantitative real-time PCR (q-PCR)

Total RNA was extracted from the cells and tissues using Trizol reagent (Invitrogen, CA, USA), according to the manufacturer’s instructions. cDNA was synthesised by M-MLV reverse transcriptase (Invitrogen) from 5 ug of total RNA. Quantitative RT-PCR was performed on the Bio-rad CFX96 real-time PCR System (Bio-rad, Foster City, CA,USA) using KAPA PROBE FAST qPCR Kits (Kapa Biosystems, MA, USA) and TaqMan probes (Invitrogen) with the following cycling conditions: 95 °C for 10 min (initial denature); then 40 cycles of 95 °C for 15 sec, 60 °C for 60 sec. The sequences of VDAC1 primers used were as follows: 5′-TGACCCTGACGCCTGCTTCT-3′, 5′-GCCACCAGCATTGACGTTCTT-3′. We used human colon cancer cell line HCT-116 as controls.

### Immunoblot analysis

For immunoblot analysis, cells were grown on 6-well plates and processed as described previously^30^. The primary antibodies were applied overnight at 4 °C, and the secondary antibodies were added for 1 h at room temperature. The VDAC1 antibody(Santa Cruz Biotechnology, sc-390996) was diluted 1:1000. The GAPDH antibody was diluted 1:5000 to verify equal loading.

### Transient transfection

Cells were transfected with specific VDAC1 siRNA oligonucleotides (Qiagen, Shanghai, China; final concentration: 20 nM) using HiPerFect transfection reagent according to the manufacturer’s instructions. The VDAC1 target sequences were as follows: 5′-AGCTTAAAAAAGTGACGGGCAGTCTGGAATCTCTTGAATTCCAGACTGCCCGTCACTG-3′ and 5′-GATCCAGTGACGGGCAGTCTGGAATTCAAGAGATTCCAGACTGCCCGTCACTTTTTTA-3′. The experiments were repeated at least three times. The effect of silencing was verified at the protein level.

### Cell Growth Assays

Cells were seeded after transfection with siRNA for the indicated time in triplicate in 96-well plates. To assess growth, the cells were kept under standard conditions for 24, 48 and 72 hours. 3-(4,5-dimethyltriazol-2-yl)-2,5-diphenyltetrazolium bromide (MTT) was added for 4 h (5 mg/ml 20 μl). Formazan products were solubilized with acidic isopropanol, and the optical density was measured at 570 nm. The growth assays were repeated three times and reported as percent changes compared to the control. PBS was used as controls.

### Immunohistochemical Analysis

Semiquantitative Immunohistochemistry (IHC) was performed to localize VDAC1 expression in 10 normal pancreas and 70 pancreatic cancer samples according to a previously published standard protocol^31^. The VDAC1 antibody was diluted at 1:5000. Two pathologists who specialised in pancreatic cancer independently rated the staining intensity and percentage of stained cells. Briefly, scores were applied to rate staining intensity in the cancer cells (no staining: 0; weak: 1; moderate: 2; strong: 3) and to determine the percentage of stained cells (<5%: 0; 5–25%: 1; >25–50%: 2; >50–75%:3; >75%:4). The final intensity score was equal to the staining intensity multiplied by the cell percentage. The staining was stratified accordingly into low levels of expression (scores 1–3) or high levels of expression (score ≥ 4).

### Invasion and Migration Assay

Invasion and migration were measured using transwell assays. Briefly, cells were seeded in 6-well plates and cultured in BD BioCoat Matrigel Invasion Chambers (BD company) at 37 °C in 5% CO2 for 48 h according to the manufacturer’s instruction. 8.0 μm membrane matrix (MILLIPORE, Darmstadt, Germany) was used to evaluate the ability of cell migration. Three parallel cultures were measured to determine the cell invasion and migration ratios of five randomly selected microscopic fields at 200X.

### Wound healing migration assay

A total of 1 × 10^6^ cells were seeded onto a 6-well plate and allowed to reach full confluence. Wounds were created by scraping confluent cell monolayers with a 1 ml pipette tip. The cells were rinsed twice to remove cellular debris. Three digital images were taken at times 0, 24 and 48 h after wounding. The assays were performed in triplicate and repeated three times. The percentage (%) change in migration was determined via comparison of the differences in wound width. Image J software(Broken Symmetry Software) was using to analyze the wound area at different time, as height was fixed, then the mean width was variable with wound area.

### Statistical Analysis

Statistical analyses were performed using SPSS 18.0. Median values were taken as cut-off limits when two groups were compared. Survival analyses were performed using the Kaplan-Meier method log-rank test. Multivariable analysis was performed using a Cox proportional hazards model. The median survival and estimations of hazard ratios were reported with 95% confidence intervals. Comparisons of demographic and clinicopathologic data between groups were made using a chi-square test. The measurement data are presented as the mean ± standard deviation (SD) and were compared using either Student’s t test or the Mann-Whitney U test. Statistical significance was set at a P value of less than 0.05. Graph-Pad Prism 4 (GraphPad, San Diego, CA) software was used to present graphs.

## Additional Information

**How to cite this article**: Wang, W. *et al.* A Single Talent Immunogenic Membrane Antigen and Novel Prognostic Predictor: voltage-dependent anion channel 1 (VDAC1) in Pancreatic Cancer. *Sci. Rep.* **6**, 33648; doi: 10.1038/srep33648 (2016).

## Supplementary Material

Supplementary Information

## Figures and Tables

**Table 1:** Positive Dots MALDI and PMF Identification of Pancreatic Cancer.

No.	Sequence (GenBank)	Name	Mw (KD)	PI	Score
1	gi|48146045	VDAC2	30849	7.49	139
2	gi|4507879	VDAC1	30868	8.63	203
3	gi|34783950	VDAC3	30981	8.85	75
4	gi|54303910	aging-associated gene 9 protein	36197	8.57	96
5	gi|6005854	prohibitin 2	33276	9.83	73
6	gi|46360168	prohibitin	29859	5.57	158
7	gi|21595242	ATP synthase, H^+^ transporting, mitochondrial F0 complex, subunit d	15820		107
8	gi|33990955	COMT protein	20374		86
9	gi|55961619	chloride intracellular channel 1	26509		84

**Table 2:** Relationship between VDAC1 expression and clinicopathological features in PDAC patients.

Variables	VDAC1 weak expression	VDAC1 strong expression	Mann–Whitney T-Test	Chi-Square
No. of patients	39	31		
Male vs. female	26/13	17/14		1.35
Age (years)	Median = 64.6	Median = 66.6	NS	
T1 & T2 vs. T3a	3/36	0/31		Ns
N0 vs. N1a	6/33	3/28		0.573
G1a	2	2		
G2	21	18		
G3	16	11		
G1 & G2 vs. G3	23 vs. 16	20 vs. 11		Ns

Ns: Not significant.

**Table 3:** Univariable and Multivariable Analysis of Prognostic Factors.

	Univariable analysis					Multivariable analysis		
	comparator	N	median survival (95% CI) in months	Log rank (X2)	p-value	HR	95% CI	p-value
Median age (years)	≥65.5 vs. <65.5	35/32	24.3 (17.4–31.2) vs. 20.2 (7.0–33.5)	0.392	0.531	1.758	0 0.908–3.402	0.094
Gender	M/F	42/25	21.5 (12.0–31.0)vs. 26.1 (18.1–34.1)	0.447	0.504	0.742	0.375–1.470	0.393
T-status	T3 vs. T1 & T2	3/64	18.1 (4.4–31.7) vs. 22.8 (16.3–29.3)	0.517	0.472	1.509	0.191–11.898	0.696
N-status	N1 vs. N0	9/58	7.4 (5.5–12.9)vs. 20.2 (11.0–29.4)	6.272	0.012	5.375	1.225–23.584	0.026
Grade	G1vs.G2 & G3	4/63	28.5 (24.5–32.5)vs.17.2 (12.7–21.7)	4.877	0.027	2203252	0.896–2.431	0.98
VDAC1 score	<4 vs. ≥4	38/29	27.0 (19.1–34.9) vs. 17.8 (8.5–27.1)	4.239	0.039	1.544	0.794–3.0	0.021
