# Supplementary material for: A Single Talent Immunogenic Membrane Antigen and Novel Prognostic Predictor: voltage-dependent anion channel 1 (VDAC1) in Pancreatic Cancer
Source: Sci Rep. 2016 Sep 23;6:33648. doi: 10.1038/srep33648 (PMC5034259; doi:10.1038/srep33648)
Supplement: Supplementary Information [file srep33648-s1.pdf]

Title: A Single Talent Immunogenic Membrane Antigen and Novel Prognostic Predictor: voltage-dependent anion channel 1 (VDAC1) in Pancreatic Cancer

Authors: Weibin Wang, Taiping Zhang, Wenjing Zhao, Lai Xu, Yu Yang, Quan Liao, Yupei Zhao

Supplement data of 9 pancreatic cancer patients' characteristics

**Variables**	No. of patients
Male	4
Female	5
Age (years)	Median = 60.3
T1& T2	3
T3a	6
N0	2
N1a	7
G1a	0
G2	4
G3	5
